# Low-density lipoprotein cholesterol levels are positively associated with the risk of endobronchial biopsy-induced refractory hemorrhage in patients with lung cancer

**DOI:** 10.1186/s12944-019-1140-2

**Published:** 2019-11-04

**Authors:** Saibin Wang, Xianqing Hu, Yibin Pan

**Affiliations:** 1 Department of Respiratory Medicine, Jinhua Municipal Central Hospital, Jinhua Hospital of Zhejiang University, No. 365, East Renmin Road, Jinhua, 321000 Zhejiang Province China; 2 Department of Cardiovascular Medicine, Jinhua Municipal Central Hospital, Jinhua Hospital of Zhejiang University, No. 365, East Renmin Road, Jinhua, 321000 Zhejiang Province China

**Keywords:** Low-density lipoprotein cholesterol, Lung cancer, Bronchoscopy, Biopsy, Hemorrhage

## Abstract

**Background:**

Lipoprotein concentrations have been associated with the major risk of bleeding events. However, whether plasma levels of LDL-C are associated with the risk of biopsy-related endobronchial hemorrhage remain elusive. Therefore, the present study was initiated to investigate the explicit association of low-density lipoprotein cholesterol (LDL-C) with endobronchial biopsy (EBB)-induced refractory hemorrhage in patients with lung cancer.

**Methods:**

This retrospective study included a total of 659 consecutive patients with lung cancer who had undergone EBB at a tertiary hospital between January 2014 and April 2018. Using multiple regression analysis, the association between LDL-C and the risk of EBB-induced refractory hemorrhage was assessed after adjusting for potential confounding factors.

**Results:**

A significant proportion (13.8%, 91/659) of the patients experienced refractory hemorrhage following EBB. In multivariate regression analysis, higher plasma LDL-C concentrations were associated with increased risk of EBB-induced refractory hemorrhage in patients with lung cancer after adjusting for potential confounders (*P* < 0.05). Using the lowest quartile of plasma LDL-C as the reference group, the odds ratio (95% confidence interval) of Q2, Q3, and Q4 were 2.32 (1.07, 5.03), 2.37 (0.94, 5.95), and 3.65 (1.16, 11.51), respectively (*P* for trend < 0.05). Moreover, this association was noticeably more pronounced in male patients with lung cancer in the subgroup analysis (*P* < 0.05).

**Conclusions:**

Plasma LDL-C was positively correlated with the increased risk of EBB-induced refractory hemorrhage in patients with lung cancer; predominantly, the associated risk was more pronounced in male patients with lung cancer.

## Background

Endobronchial biopsy (EBB) via flexible bronchoscopy has emerged as a first-line minimally invasive transbronchial biopsy modality in the histopathologic diagnosis of pulmonary diseases [1]. Usually, the procedure has been proven to be safe; however, significant hemorrhage or refractory bleeding is frequently encountered by bronchoscopists. Moreover, massive biopsy-related endobronchial hemorrhage in the airway remains most difficult-to-manage complication and could be life-threatening due to difficult hemostasis following EBB [2, 3].

Lipoprotein concentrations have been associated with the risk of major bleeding in the brain and airways [4–6]. Low-density lipoprotein cholesterol (LDL-C) represents a well-established risk factor for cardiovascular disease [7] and has been documented to be associated with high risk of intracerebral hemorrhage [8, 9]. However, whether plasma levels of LDL-C are associated with the risk of biopsy-related endobronchial hemorrhage remain elusive. As refractory bleeding during bronchoscopy remains extremely challenging and there are no definitive preoperative predictors reported, the present study hypothesized that LDL-C may be associated with biopsy-related endobronchial hemorrhage and may represent a potentially modifiable risk factor prior to EBB. Predominantly, patients with lung cancer are subjected to EBB, and malignant lesions were considered to be more susceptible to hemorrhage than benign mucosal lesions during EBB [10]. Herein, the present study was initiated to investigate the explicit relationship between plasma LDL-C and EBB-induced refractory hemorrhage in patients with lung cancer.

## Methods

### Study design and subjects

This retrospective cohort study included a total of 659 consecutive patients with lung cancer who underwent EBB at a large tertiary hospital (Jinhua Municipal Central Hospital, Jinhua, China) between January 2014 and April 2018. Patients’ inclusion criteria were as follows: a. adult patients with endobronchial local exophytic lesions who had undergone EBB; b. histopathologically confirmed a diagnosis of primary lung cancer. The patients were excluded if presented any of the following risk factors, platelet count < 50 × 10^9^/L, coagulation dysfunction, bleeding tendencies, receiving continuous anti-platelet therapy or continuous anticoagulant therapy, presented with severe liver or kidney disease, heart failure, uncontrolled hypertension or pulmonary arterial hypertension, on mechanical ventilation, and on immunosuppressive status. Patients receiving antiplatelet drugs were asked to withhold medicines one week prior to bronchoscopy. For this study, refractory hemorrhage was defined as the EBB-induced hemorrhage with failed hemostasis requiring intrabronchial instillation of hemostatic drugs (4 °C physiological saline and diluted (1:10000) adrenalin), and thus additional hemostatic measures (argon plasma coagulation (APC), electrocoagulation, vasopressin or hemocoagulase) were requisite for hemostasis [11]. Patients were classified into the EBB-induced refractory hemorrhage group and the non-refractory hemorrhage group based on hemostatic measures applied following EBB. This study complies with the Declaration of Helsinki and was approved by the ethics committee of Jinhua Municipal Central Hospital. Informed consent was waived because the data used in this study were anonymous.

### Variables collection

The following clinicopathological characteristics of patients were retrieved from the electronic medical record, including gender, age, smoking history, weight, systolic blood pressure (SBP), diastolic blood pressure, location of the lesion, histological type, and stage of cancer. Data on patient’s comorbidities including hypertension, chronic obstructive pulmonary disease (COPD), coronary heart disease (CHD), and diabetes mellitus were also collected. Laboratory investigations data including LDL-C, high-density lipoprotein cholesterol (HDL-C), total cholesterol, triglyceride, apolipoprotein B, apolipoprotein E, platelet counts, white blood cell counts, neutrophil percentage, hemoglobin, prothrombin time (PT), activated partial thromboplastin time (APTT), C-reactive protein (CRP), aspartate aminotransferase, and alanine aminotransferase (ALT) were also recorded. Blood laboratory investigations were performed on the first visit or admission, three days prior to EBB.

### EBB procedures

All bronchoscopic and EBB procedures were performed by two experienced bronchoscopists. The procedures followed were as described previously [11]. Three to five biopsies were collected from the same endobronchial exophytic lesion using rigid endoscopic biopsy forceps [12]; however, when the lesion bled significantly following the first biopsy, only one biopsy was conducted. In this study, EBB-induced hemorrhage was self-limiting or hemostasis achieved using hemostatic drugs, including intrabronchial instillation of cold physiological saline (4 °C) and/or diluted adrenalin, intravenous injection of vasopressin and/or hemocoagulase, electrocoagulation, and APC.

### Statistical analysis

Descriptive statistics were used to summarize clinicopathological characteristics. Categorical variables are expressed as the number (percentage) and continuous variables as the median (interquartile). Unpaired *t*-tests or Mann-Whitney U test, Pearson chi-squared tests or the Fisher’s exact, were used for the comparison between the two groups, as appropriate. The correlation between LDL-C level and the risk of EBB-induced refractory hemorrhage was examined using the smoothing curve fitting via a generalized additive model. Multivariate regression analysis was performed to evaluate the independent effects of LDL-C on the risk of EBB-induced refractory hemorrhage with or without adjustment for potential confounders. Two criteria for adjusting potential confounders were applied in this study: (1) model I: variables that needed to be adjusted by clinical significance [5, 6, 13]; (2) model II: in covariant-discrimination algorithm, variables included producing a change ≥10% of the regression coefficient after introduction into the basic model or removing from the complete model; and the regression coefficient of co-variable to dependent variable yielded *P*-value < 0.05. All analyses were performed using R software (version 3.5.3), and *P*-value < 0.05 was considered statistically significant.

## Results

**Fig. 1:**
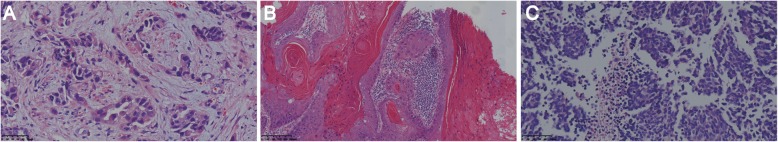
Microscopic appearance (H&E). Histological types of lung adenocarcinoma (**a**, × 400), lung squamous cell carcinoma (**b**, × 100), and small-cell lung carcinoma (**c**, × 400)

The clinicopathological characteristics and laboratory investigation of the patients were summarized in Table 1. A total of 91 (13.8%) of the patients with lung cancer experienced refractory bleeding following EBB; however, no case of death due to severe bleeding was recorded. The median levels of plasma LDL-C of all the subjects were 2.8 mmol/L. There was no significant difference in the levels of plasma LDL-C between the refractory hemorrhage group and the non-refractory hemorrhage group (*P* > 0.05, Table 1). Location of the lesion, histological type (Fig. 1), neutrophil percentage, CRP, PT, APTT, and ALT correlated with EBB-induced refractory bleeding as assessed by univariate analysis (*P* < 0.05, Table 1).

**Table 1:** Baseline characteristics of the study participants

Variables	EBB-induced refractory hemorrhage		*P*-value
	Yes (*n* = 91)	No (*n* = 568)	
Gender, *n* (%)			0.305
Female	16 (17.6)	127 (22.4)	
Male	75 (82.4)	441 (77.6)	
Age (year), median (Q1-Q3)	65 (59–72)	65 (59–70)	0.561
Weight (kg), median (Q1-Q3)	59.5 (53.0–65.2)	59.0 (53.0–66.0)	0.794
Smoking, *n* (%)			0.027
No	24 (26.4)	218 (38.4)	
Yes	67 (73.6)	350 (61.6)	
SBP (mmHg), median (Q1-Q3)	129 (112–144)	131 (119–145)	0.285
DBP (mmHg), median (Q1-Q3)	79.0 (71–88)	78.0 (70–87)	0.361
Coexisting Disease			
COPD, *n* (%)			0.826
No	84 (92.3)	528 (93.0)	
Yes	7 (7.7)	40 (7.0)	
Hypertension, *n* (%)			0.140
No	63 (69.2)	434 (76.4)	
Yes	28 (30.8)	134 (23.6)	
Diabetes mellitus, *n* (%)			0.294
No	89 (97.8)	538 (94.7)	
Yes	2 (2.2)	30 (5.3)	
CHD, *n* (%)			1.000
No	88 (96.7)	548 (96.5)	
Yes	3 (3.3)	20 (3.5)	
Tumor Characteristics			
Location of the lesion, *n* (%)			0.045
Central airways	20 (22.0)	79 (13.9)	
Peripheral bronchi	71 (78.0)	489 (86.1)	
Stage of cancer, *n* (%)			0.461
Early	52 (57.1)	301 (53.0)	
Advanced	39 (42.9)	267 (47.0)	
Histological types, *n* (%)			< 0.001
Adenocarcinoma	11 (12.1)	165 (29.0)	
Squamous cell carcinoma	63 (69.2)	268 (47.2)	
SCLC	13 (14.3)	103 (18.1)	
Other^a^	4 (4.4)	32 (5.6)	
Laboratory Tests, median (Q1-Q3)			
WBC (×10^9^/L)	6.8 (5.5–8.9)	6.8 (5.5–8.6)	0.977
Neutrophils (%)	72.2 (64.2–80.4)	69.5 (61.9–76.1)	0.025
Hemoglobin (g/L)	126 (116–137)	128 (116–140)	0.325
platelets (× 10^9^/L)	237 (167–296)	225 (174.0–284)	0.687
CRP (mg/L)	12.7 (3.0–37.7)	5.9 (1.1–28.9)	0.009
PT (S)	13.2 (12.3–13.6)	12.6 (11.7–13.3)	< 0.001
APTT (S)	36.1 (33.0–40.0)	34.0 (31.5–37.1)	< 0.001
ALT (IU/L)	14.0 (11.5–21.3)	17.0 (12.0–26.0)	0.027
AST (IU/L)	22.6 (18.0–27.1)	23.0 (19.0–29.0)	0.289
Triglyceride (mmol/L)	1.0 (0.8–1.4)	1.1 (0.8–1.5)	0.793
TC (mmol/L)	4.1 (3.6–4.7)	4.1 (3.5–4.8)	0.663
HDL-C (mmol/L)	1.1 (0.9–1.2)	1.1 (0.9–1.3)	0.069
LDL-C (mmol/L)	2.9 (2.4–3.4)	2.7 (2.2–3.3)	0.143
Apolipoprotein B (g/L)	1.0 (0.8–1.2)	1.0 (0.8–1.2)	0.569
Apolipoprotein E (mg/dL)	3.3 (2.5–4.4)	3.6 (2.8–4.7)	0.078

^a^ “Other” includes histological type of NSCLC (not specified, *n* = 12), neuroendocrine carcinoma (*n* = 7), adenosquamous carcinoma (*n* = 5), muco-epidermoid carcinoma (*n* = 2), adenoid cystic carcinoma (*n* = 1), carcinosarcoma (*n* = 1) and lung cancer without histological type specified (*n* = 8). EBB, endobronchial biopsy, SBP, systolic blood pressure, DBP, diastolic blood pressure, COPD, chronic obstructive pulmonary disease, CHD, coronary heart disease, SCLC, small-cell lung carcinoma, WBC, white blood cell, CRP, C-reactive protein, PT, prothrombin time, APTT, activated partial thromboplastin time, ALT, alanine aminotransferase, AST, aspartate aminotransferase, TC, total cholesterol, HDL-C, high-density lipoprotein cholesterol, LDL-C, low-density lipoprotein cholesterol

**Fig. 2:**
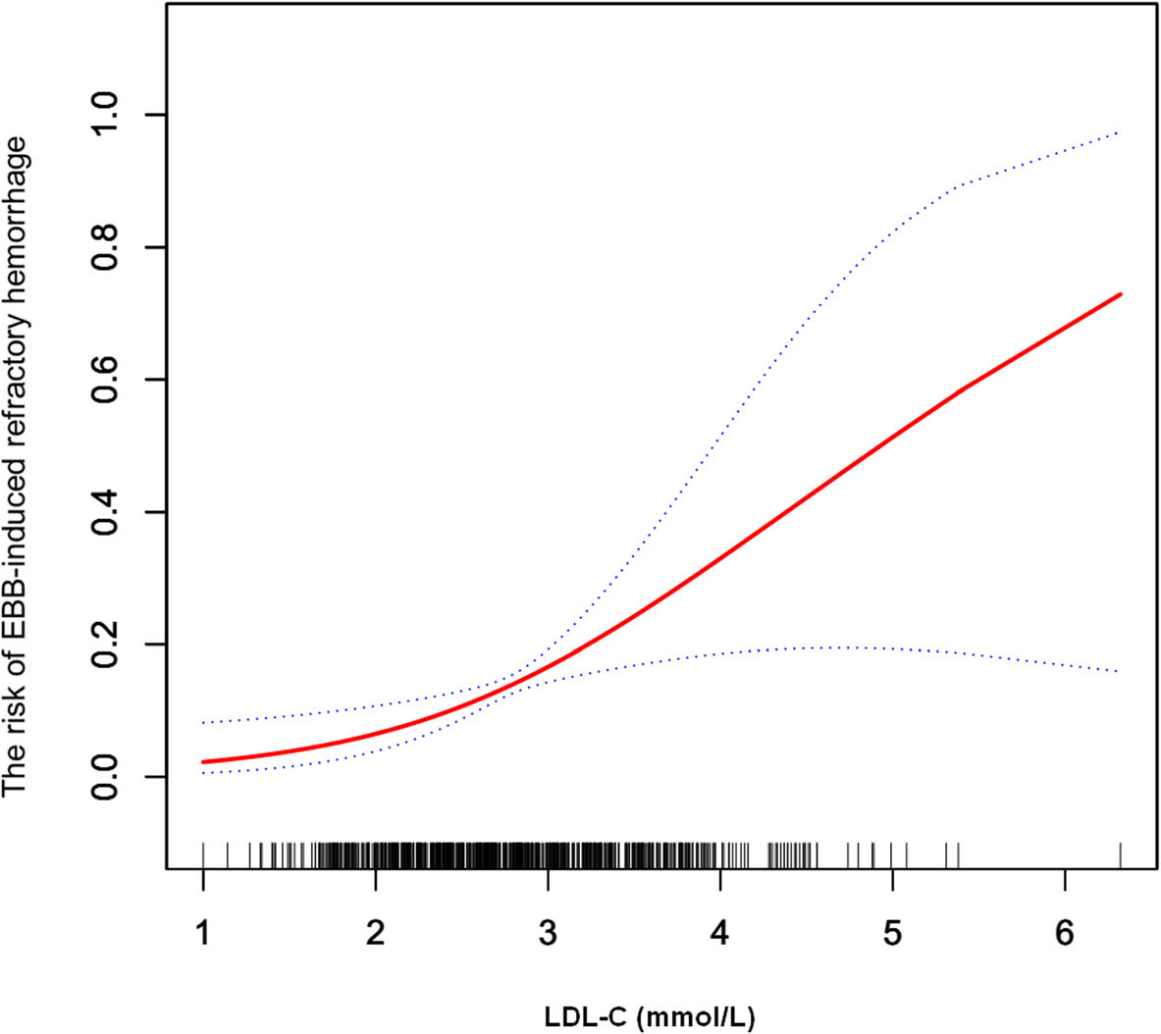
The smooth curve fitting shows the association between plasma LDL-C and the risk of EBB-induced refractory hemorrhage after adjusting for the main confounders (gender, location of the lesion, histological type, smoking, SBP, hemoglobin, neutrophil percentage, PT, APTT, CRP, ALT, triglyceride, HDL-C, apolipoprotein B, and apolipoprotein E). Dotted lines represented the upper and lower 95% confidence intervals. LDL-C, low-density lipoprotein cholesterol; EBB endobronchial biopsy; SBP, systolic blood pressure; PT, prothrombin time; APTT, activated partial thromboplastin time; CRP, C-reactive protein; ALT, alanine aminotransferase; HDL-C, high-density lipoprotein cholesterol

Smoothing curve fitting showed a significant correlation between plasma levels of LDL-C and the risk of EBB-induced refractory hemorrhage (Fig. 2). Increased risk of bleeding with increasing levels of LDL-C was observed in multivariate regression analyses after adjusting for the potential confounders either in the model I (adjusted for location of the lesion, histological type, stage, HDL-C, and apolipoprotein E) or in model II (adjusted for gender, location of the lesion, histological type, smoking, SBP, hemoglobin, neutrophil percentage, PT, APTT, CRP, ALT, triglyceride, HDL-C, apolipoprotein B, and apolipoprotein E) (Table 2). Furthermore, using the lowest quartile of plasma LDL-C as the reference group, the odds ratio (95% confidence interval [CI]) of Q2, Q3, and Q4 were 2.32 (1.07, 5.03), 2.37 (0.94, 5.95), and 3.65 (1.16, 11.51) (*P* for trend < 0.05, Table 2).

**Table 2:** Multivariate regression analysis of LDL-C and the risk of EBB-induced refractory hemorrhage

Parameter	Crude OR (95% CI) *p*-value	Model I OR (95% CI) *p*-value	Model II OR (95% CI) *p*-value
LDL-C (mmol/L)	1.24 (0.93, 1.65) 0.1437	1.48 (1.08, 2.02) 0.0142	2.65 (1.40, 5.00) 0.0026
Quartile of LDL-C (mmol/L)			
Q1 (< 2.26)	Ref.	Ref.	Ref.
Q2 (2.26–2.75)	1.81 (0.93, 3.55) 0.0830	2.01 (1.01, 4.01) 0.0465	2.32 (1.07, 5.03) 0.0327
Q3 (2.76–3.28)	1.57 (0.78, 3.13) 0.2031	1.82 (0.89, 3.71) 0.1011	2.37 (0.94, 5.95) 0.0665
Q4 (> 3..28)	1.73 (0.88, 3.41) 0.1110	2.48 (1.21, 5.07) 0.0128	3.65 (1.16, 11.51) 0.0270
P for trend	0.2013	0.0259	0.0464

Model I adjusts for location of the lesion, histological types, stage of cancer, HDL-C, and apolipoprotein E. Model II adjusts for gender, location of the lesion, histological type, smoking, SBP, hemoglobin, neutrophil percentage, PT, APTT, CRP, ALT, triglyceride, HDL-C, apolipoprotein B, and apolipoprotein E. LDL-C, low-density lipoprotein cholesterol; EBB endobronchial biopsy; SBP, systolic blood pressure; PT, prothrombin time; APTT, activated partial thromboplastin time; CRP, C-reactive protein; ALT, alanine aminotransferase; HDL-C, high-density lipoprotein cholesterol

In subgroup analyses, a significant association was observed for gender (*P* for interaction = 0.0256, Table 3); however, there was no statistical significance observed for smoking, coexisting diseases (hypertension, diabetes mellitus, CHD, and COPD), location of the lesion, stage of cancer, and histological type (*P* for interaction > 0.05). These results indicated that there was a significant association between gender of patients and plasma LDL-C. Precisely, the effect of LDL-C on the risk of EBB-induced refractory hemorrhage in patients with lung cancer exhibited a significant difference between the two genders. Significantly higher plasma levels of LDL-C were observed in male patients (β = 3.81 (95% CI: 1.88, 7.72)) with EBB-induced refractory hemorrhage than female patients (β = 0.60 (95% CI: 0.12, 2.91)).

**Table 3:** Effect of LDL-C on EBB-induced refractory hemorrhage in subgroups

Characteristic	Number of patients	β(95%CI)	P for interaction
Gender			0.0256
Female	143	0.60 (0.12, 2.91)	
Male	516	3.81 (1.88, 7.72)	
Smoking			0.6460
Yes	242	2.86 (1.38, 5.96)	
No	417	2.12 (0.68, 6.60)	
Hypertension			0.8006
Yes	162	2.46 (1.08, 5.60)	
No	497	2.68 (1.39, 5.19)	
Diabetes mellitus			0.9991
Yes	32	0.00 (0.00, inf)	
No	627	2.75 (1.44, 5.27)	
CHD			0.9994
Yes	23	inf. (0.00, inf)	
No	636	2.59 (1.33, 5.05)	
COPD			0.5498
Yes	612	5.73 (0.41, 79.26)	
No	47	2.65 (1.36, 5.14)	
Location of the lesion			0.6326
Peripheral bronchi	560	2.65 (1.31, 5.35)	
Central airway	99	3.84 (0.94, 15.58)	
Stage			0.7970
Early	353	2.76 (1.27, 5.99)	
Advanced	306	2.38 (0.93, 6.10)	
Histological type			0.1136
Adenocarcinoma	176	0.37 (0.05, 2.48)	
Squamous cell carcinoma	331	4.61 (1.98, 10.71)	
SCLC	116	2.85 (0.48, 16.94)	
Other^a^	36	inf. (0.00, inf)	

Adjusted for gender, location of the lesion, histological type, smoking, SBP, hemoglobin, neutrophil percentage, PT, APTT, CRP, ALT, triglyceride, HDL-C, apolipoprotein B, and apolipoprotein E; in each case, the model was not adjusted for the stratification variable. ^a^“Other” includes histological types of NSCLC (not specified, *n* = 12), neuroendocrine carcinoma (*n* = 7), adenosquamous carcinoma (*n* = 5), muco-epidermoid carcinoma (*n* = 2), adenoid cystic carcinoma (*n* = 1), carcinosarcoma (*n* = 1) and lung cancer without histological type specified (*n* = 8). LDL-C, low-density lipoprotein cholesterol; EBB endobronchial biopsy; CHD, coronary heart disease; COPD, chronic obstructive pulmonary disease; NSCLC, non-small cell lung carcinoma; SBP, systolic blood pressure; PT, prothrombin time; APTT, activated partial thromboplastin time; CRP, C-reactive protein; ALT, alanine aminotransferase; HDL-C, high-density lipoprotein cholesterol

## Discussion

The present study investigated levels of plasma LDL-C in patients with endobronchial local exophytic lesions who underwent EBB for its possible relationship with EBB-induced refractory hemorrhage in patients with lung cancer. The results indicated that levels of plasma LDL-C were positively correlated with EBB-induced refractory hemorrhage in patients with lung cancer. Furthermore, there was a significant association between gender of patients with lung cancer and levels of plasma LDL-C, implying that male patients with lung cancer had a significantly higher risk of refractory bleeding than female patients following EBB.

EBB-induced hemorrhage is the most frequent and difficult-to-manage complication encountered during bronchoscopy, particularly when performing biopsies in patients with lung cancer. Conceivably, endobronchial refractory bleeding or massive hemorrhage in the airway may be life-threatening [2, 3]. Several factors have been associated with the risk of bleeding during bronchoscopy, including immunosuppressive status, thrombocytopenia (platelet count < 50 × 10^9^/L), uncontrolled hypertension or pulmonary arterial hypertension, lung transplant, anticoagulant and/or antiplatelet drug use, severe liver and/or kidney disease, and bleeding tendencies [6, 13–16]. In recent years, accumulating evidence has suggested that high concentrations of lipoprotein(a) have been associated with low risk of major bleeding episodes in the brain and airways [4]. With regard to intra-airway intervention-related bleeding, HDL-C and apolipoprotein-E were noticeably associated with the risk of EBB-induced bleeding in a non-linear pattern [5, 6]. Consistently, this study found that elevated LDL-C level was correlated with increased risk of EBB-induced refractory hemorrhage. Moreover, this association between the two did not lose its significance even after strict statistical adjustments for potential confounders. Although the exact mechanism underlying this association remains undetermined, it may be mediated partially through the regulation of adiponectin, an adipose-derived cytokine, which has been reported to exhibit an effect on platelet hyperactivity, hypercoagulability, and hypofibrinolysis [17–19]. Apparently, there was an inverse relationship between blood adiponectin and LDL-C [20], and LDL-C could alter DNA methylation levels of adiponectin both in adipose tissues and blood cells [21].

Furthermore, in this study, we performed subgroup analyses and interaction tests. An interaction effect was observed between gender and LDL-C, which indicated that LDL-C levels had a greater impact on male patients with lung cancer and caused refractory hemorrhage following EBB than on female patients. This finding might help recognize patients at increased risk of developing hemorrhage in a preoperative individualized assessment of bleeding risk, and provide evidence for gender-related differences in LDL-C management in patients with lung cancer [22].

Several limitations of the present study are worth noting. Firstly, this was a single-center retrospective study. Although we performed stringent statistical adjustments to minimize residual confounding factors, the study inevitably suffered from confounders and selection bias. Moreover, subjects included in this study were divided into the refractory bleeding group or non-refractory bleeding group based on hemostasis measures they received following EBB, and thus this classification might have introduced biases arising from an inaccurate grouping of some patients. Secondly, in patients with refractory hemorrhage, there was a high probability of difference in the amount of bleeding; however, we could not provide a quantitative measurement of blood loss. Besides, it remains challenging to accurately quantify blood loss during bronchoscopy [23]. Despite the aforementioned limitations, this study was the first to reveal the explicit relationship between plasma LDL-C and the risk of EBB-induced refractory hemorrhage in patients with lung cancer.

## Conclusions

In conclusion, the findings of the present study indicated a positive correlation of plasma LDL-C with the increased risk of EBB-induced refractory bleeding in patients with lung cancer, and this associated risk was more pronounced in male patients. Furthermore, this finding might assist clinicians in recognizing patients with increased risk of EBB-induced hemorrhage prior to bronchoscopy for their possible improved management during EBB.

## Data Availability

The datasets used and/or analyzed during the current study are available from the corresponding author on reasonable request.

## Abbreviations

ALT: Alanine aminotransferase
APC: Argon plasma coagulation
APTT: Activated partial thromboplastin time
CHD: Coronary heart disease
CI: Confidence interval
COPD: Chronic obstructive pulmonary disease
CRP: C-reactive protein
EBB: Endobronchial biopsy
HDL-C: High-density lipoprotein cholesterol
LDL-C: Low-density lipoprotein cholesterol
PT: Prothrombin time
